# Evaluating digital medicine ingestion data from seriously mentally ill patients with a Bayesian Hybrid Model

**DOI:** 10.1038/s41746-019-0095-z

**Published:** 2019-03-22

**Authors:** Jonathan Knights, Zahra Heidary, Timothy Peters-Strickland, Murali Ramanathan

**Affiliations:** 1 Otsuka Pharmaceutical Development & Commercialization, Inc.: 508 Carnegie Center, Princeton, NJ USA; 2 State University of Buffalo at New York, School of Pharmacy and Pharmaceutical Sciences, 355 Kapoor Hall, Buffalo, NY 14214 USA

**Keywords:** Translational research, Outcomes research

## Abstract

The objective of this work was to adapt and evaluate the performance of a Bayesian hybrid model to characterize objective temporal medication ingestion parameters from two clinical studies in patients with serious mental illness (SMI) receiving treatment with a digital medicine system. This system provides a signal from an ingested sensor contained in the dosage form to a patient-worn patch and transmits this signal via the patient’s mobile device. A previously developed hybrid Markov-von Mises model was used to obtain maximum-likelihood estimates for medication ingestion behavior parameters for individual patients. The individual parameter estimates were modeled to obtain distribution parameters of priors implemented in a Markov chain-Monte Carlo framework. Clinical and demographic covariates associated with model ingestion parameters were also assessed. We obtained individual estimates of overall observed ingestion percent (median:75.9%, range:18.2–98.3%, IQR:32.9%), rate of excess dosing events (median:0%, range:0–14.3%, IQR:3.0%) and observed ingestion duration. The modeling also provided estimates of the Markov-dependence probabilities of dosing success following a dosing success or failure. The ingestion-timing deviations were modeled with the von Mises distribution. A subset of 17 patients (22.1%) were identified as prompt correctors based on Markov-dependence probability of a dosing failure followed by a dosing success of unity. The prompt corrector sub-group had a better overall digital medicine ingestion parameter profile compared to those who were not prompt correctors. Our results demonstrate the potential utility of a Bayesian Hybrid Markov-von Mises model for characterizing digital medicine ingestion patterns in patients with SMI.

## Introduction

Lack of adherence to medication is an important factor that contributes to increased healthcare utilization^1,2^: Among patients with serious mental illness (SMI)—which includes schizophrenia, bipolar disorder, and major depression—this is of particular concern, with some reports estimating rates of nonadherence as high as 60%.^1,3^ Within the SMI population, effective pharmacotherapy is critical for managing the risk of serious potential adverse events such as relapse of psychosis, recurrence of symptoms, poor social functioning, hospitalizations, and suicide attempts.^4,5^

Conventional methods of inferring medication ingestion adherence to pharmacotherapy are limited in their utility as they acquire data on surrogate measures associated with medication ingestion events and involve subjectivity. Examples of older methods with high subjectivity are patient self-reports, medication possession ratio,^6^ and percentage of days covered.^7^ Newer approaches such as electronic blister packs and medication event monitoring systems have lower subjectivity; however, these methods assume that the interaction with the packaging implies successful ingestions. Pharmacokinetic sampling is sometimes leveraged as an objective measure of general adherence, but is sub-optimal in routine clinical practice because it is invasive and provides only a single snapshot in what may have been many weeks of ingestion opportunities. Given the limitations of conventional medication ingestion monitoring systems, there is a clearly recognized but yet unmet clinical need that digital medicine systems are ideally suited to address.

Digital medicine, in this context, refers to the combination of an active pharmaceutical and an ingestible sensor component that communicates to a mobile or web-based application to capture that a patient has taken their medication at a specific time.^8^ A core objective of digital medicine systems is to improve patient adherence^9,10^; however, one of their primary advantages over competing alternatives is that they provide a signal corresponding to successful medication ingestion events, which can directly enable timely and impactful interventions by the care team.^10^ These systems have the potential for a transformative impact on understanding medication ingestion behaviors, which could lead to better public health outcomes over time.

Given the importance of medication ingestion data for informing clinical decision making (particularly in SMI) and the rising prevalence of risk stratification and predictive models in both the clinical and population health settings, the successful application of statistical frameworks for describing objective patient medication ingestion patterns is of value. This research applies a novel Bayesian model to characterize digital medicine data from two clinical studies in SMI: The model,^11–13^ which has not been extensively investigated in digital medicine, or in SMI patients, provides informative metrics on medication ingestion patterns including observed medication ingestion percent, excess dosing, duration of observed treatment, probability of dosing succeses following dosing successes or failures, and medication ingestion-timing deviations.

## Results

### Description of the digital medicine system

**Fig. 1:**
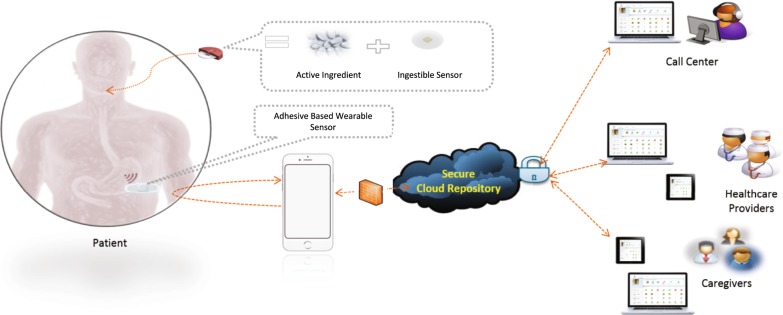
An overview of the digital medicine system (DMS). From left to right: Patient takes medication embedded with ingestible sensor, which is activated in the stomach. The ingestible sensor is detected by the wearable sensor, which sends its collected information (including additional sensors not depicted) to the patient’s smartphone. The information is then passed on to a secure cloud infrastructure where it can be made available to appropriate members of the patient’s care team

The digital medicine system leveraged in this work is composed of a wearable sensor (patch), a mobile application, and an ingestible sensor embedded in an active pharmaceutical, which has been developed to capture medication ingestions in patients with SMI^14^ (Fig. 1).

### Demographic, clinical, and dosing characteristics

The overall demographic and clinical characteristics of the study sample are summarized in Table 1. Demographic, clinical and prescribed dosing data from 79 subjects from two clinical studies, with 49 and 30 subjects, respectively were pooled. Two subjects (one from each study) were excluded from the modeling because they took only one dose.

**Table 1:** Demographic, clinical characteristics of the study sample

Sample size *n*	77
Females: Males (% Female)	36:41 (47%)
Age, years	47.0 ± 12
Race	
Black or African–American	41 (53%)
Asian	3 (4%)
White	31 (40%)
Other	2 (3%)
Ethnicity	
Hispanic or Latino	2 (3%)
Not Hispanic or Latino	75 (97%)
Diagnosis	
Schizophrenia	44 (57%)
Bipolar 1 disorder	22 (29%)
Major depressive disorder	11 (14%)
Disease duration, years	11.9 ± 11
Clinical Global Impression—Severity (CGI-S) Score^a^	3.13 ± 0.85
Personal and Social Performance Scale (PSP) Score^b^	71.8 ± 14

^a^Clinical Global Impression—Severity scale (CGI-S) is a 7-point scale measure for the severity of the patient’s illness at the time of assessment, relative to the clinician’s past experience with patients who have the same diagnosis (https://en.wikipedia.org/wiki/Clinical_Global_Impression)

^b^Personal and Social Performance Scale (PSP) measures functioning and social performance: Original reference^30^

The study sample contained representative numbers of the White–American and Black or African–American subjects. However, the numbers of Asian and Other racial groups were small as were the number of subjects of Hispanic or Latino ethnicity. There were no American Indian or Alaska Natives and Native Hawaiian or Other Pacific Islanders.

Table 2 summarizes the prescribed drug dosing regimen and observed digital medicine ingestion-related characteristics in the study sample. The median aripiprazole dose was 15 mg (Interquartile range = 10, Range: 2–30 mg). The median number of concomitant medications was 4 (Interquartile range = 3, Range: 1–12). The median duration (days) elapsed between first and last ingestion was 53 days, with a median of 75.9% of the expected ingestions being observed (observed ingestion percent). Finally, The frequency of excess dosing events ranged from 0–14.3% across patients.

**Table 2:** Drug adherence characteristics

Sample size *n*	77
Aripiprazole dose	
2 mg	7 (9%)
5 mg	8 (10%)
10 mg	23 (30%)
15 mg	14 (18%)
20 mg	10 (13%)
30 mg	15 (20%)
Number of concomitant medications	
1	9 (12%)
2	9 (12%)
3	15 (30%)
4	11 (15%)
5	9 (12%)
6	4 (5%)
7	8 (11%)
8	3 (4%)
9	5 (7%)
>10	2 (3%)
Missing data	2
Observed ingestion duration, days, median (Range, IQR)	53 (6–64, 20)
Observed ingestion percent %, median (Range, IQR)	75.9% (18.2–98.3%, 32.9%)
Observed fraction of excess dosing events (%), median (Range, IQR)	0% (0 −14.3%, 3.0%)

### Bayesian modeling of digital medicine ingestion parameters

**Fig. 2:**
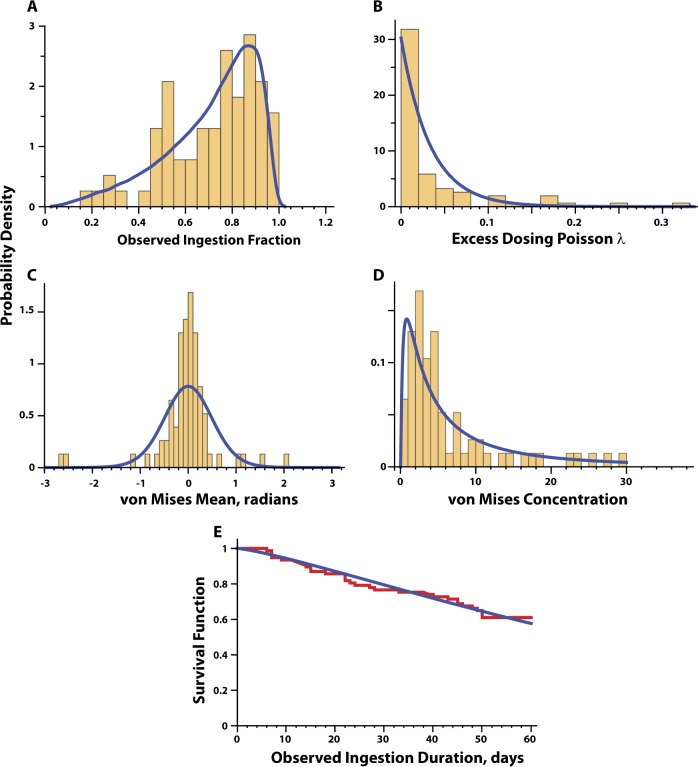
Probability density histograms for observed ingestion fraction (**a**), Poisson *λ* (**b**) of excess dosing events, von Mises mean (**c**), von Mises concentration (**d**) and the survival function of observed ingestion duration (**e**) are overlaid on their corresponding modeled distribution shown in blue lines. For reference to text, Observed ingestion fraction = observed ingestion percent/100

Figure 2 compares the observed probability density histograms for overall observed ingestion percent (OIP), the Poisson *λ* parameter describing the observed excess dosing events, as well as mean and concentration parameters for the von Mises distribution describing the observed ingestion-timing deviations. The Bayesian model satisfactorily characterized the OIP density function and the monotonically decreasing nature of the Poisson *λ* parameter density function. The fit of the von Mises concentration parameter was satisfactory but there was modest lack of fit for the sharp peak of the von Mises mean density function. We hypothesized that this deviation was potentially related to the excess density at *p*_FS_ = 1, which we address in the next section.

**Fig. 3:**
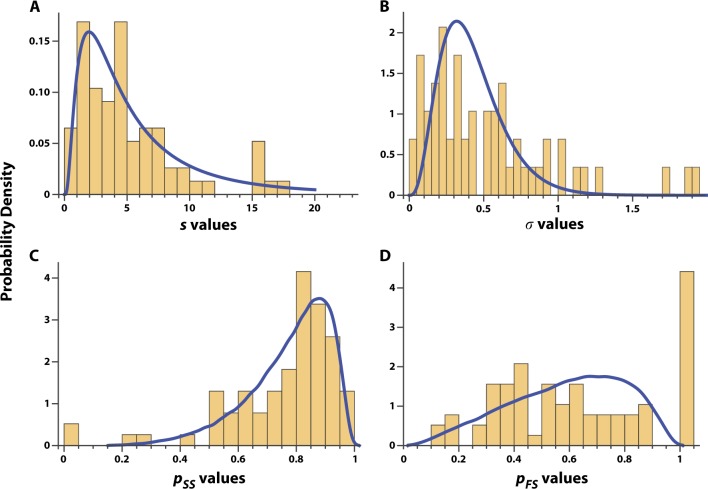
Probability density histograms of the observed values of *s* (**a**), *σ* (**b**), *p*_SS_ (**c**) and *p*_FS_ (**d**) are overlaid on their corresponding modeled distribution shown in blue lines

The two main Markov parameters, *p*_SS_ and *p*_FS_, captured the probability of an ingestion success following an ingestion success, and the probability of an ingestion success following an ingestion failure, respectively. With the exception of the aforementioned excess point density at *p*_FS_ = 1, both *p*_SS_(*i*) and *p*_*FS*_(*i*) were adequately captured as functions of the parameters *s*(*i*) and *σ*(*i*) from helix-coil theory in polymer physics^15–17^ (see Methods). Figure 3 compares the observed probability density histograms for *s*(*i*) and *σ*(*i*), as well as *p*_SS_(*i*) and *p*_FS_(*i*), to the corresponding Bayesian probability density estimates. The observed probability density histograms of all four parameters had asymmetric non-Gaussian characteristics. The long-tailed density of *s*(*i*) and *σ*(*i*), as well as the right skewed, domain-limited density of *p*_SS_(*i*) and *p*_FS_(*i*) were satisfactorily characterized by our Bayesian model priors—again, with the exception of the excess density at *p*_FS_ = 1.

### Digital medicine ingestion parameter profiles of the prompt corrector sub-group

We investigated the lack of fit at *p*_FS_ = 1 further. For purposes of these additional analyses, we defined a patient to be a Prompt Corrector (PC) if they exhibited *p*_FS_ = 1 and as a Non-Prompt Corrector (NPC) otherwise.

The proportion of PC patients was 22.1% (*N* = 17): This sub-group was comprised of 13.6% (6 of 44) of the schizophrenia sample, 36.4% (8 of 22) of the bipolar type 1 sample, and 27.3% (3 of 11) of the major depressive disorder sample. These differences did not reach statistical significance (*p* = 0.11, *χ*^2^ test, df = 5). We did not find evidence for differences in aripiprazole dose (*p* = 0.20, Mann–Whitney test), number of concomitant medications (*p* = 0.65, Mann–Whitney test), baseline Clinical Global Impression Severity scale (CGI-S, a 7-point clinician-based scale measure of disease severity, higher scores are worse) values (*p* = 0.063, Mann–Whitney test) or baseline Personal and Social Performance Scale (PSP) Score of functioning and social performance (*p* = 0.91, Mann-Whitney test). We did not obtain evidence for differences in gender (*p* *=* 0.10, Fisher Exact Test) or race (*p* = 0.42, Fisher Exact Test, for Black or African-American relative to White) distributions in the PC vs. NPC groups. Ethnicity differences were not evaluated in statistical testing because there were only 2 Hispanic or Latino subjects. The mean age of the PC (median: 52 years, IQR: 18 years, range: 19–64 years) and NPC (median: 49 years, IQR: 20 years, range: 20–62 years) groups was similar (*p* = 0.34, Mann-Whitney test). However, the PC group had a lower (*p* < 0.001, Mann–Whitney test) disease duration (median: 5.00 years, IQR: 8.00 years, range: 1–26 years) compared to the NPC group (median: 14.0 years, IQR: 16.0 years, range: 1–38 years).

**Fig. 4:**
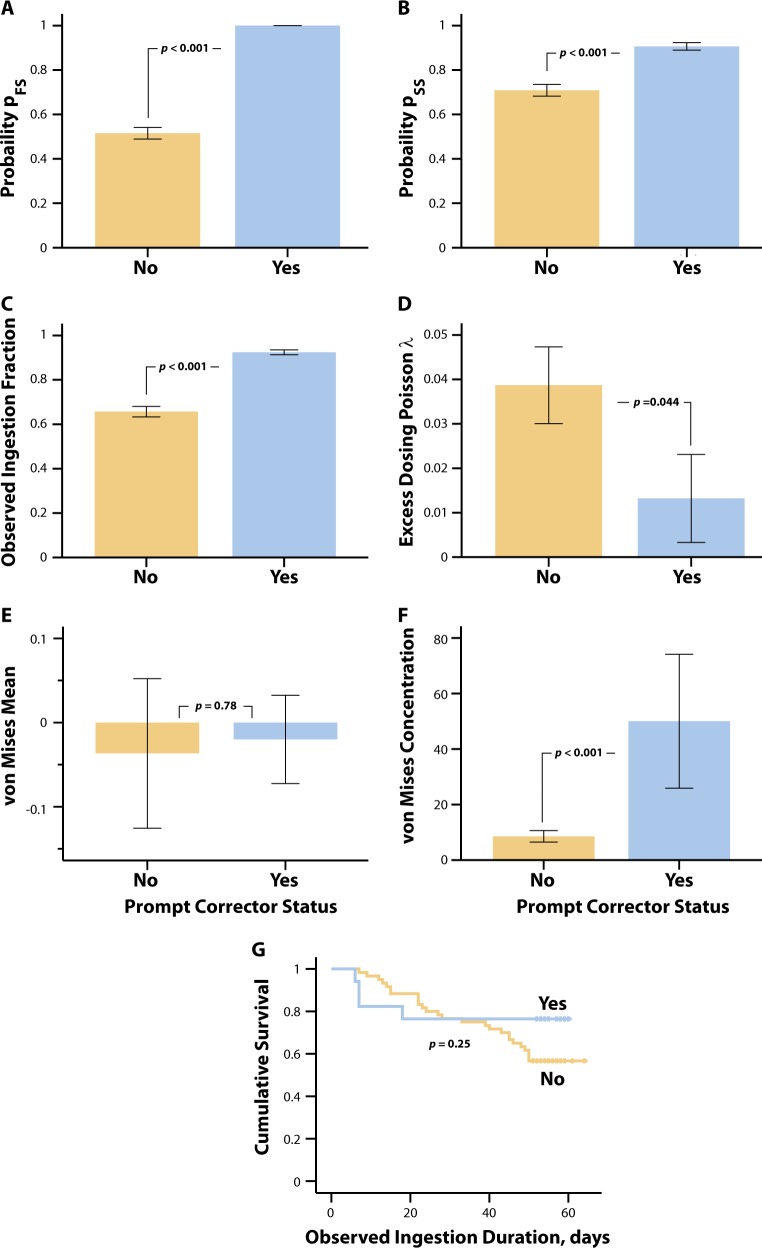
Comparison of the digital medicine ingestion parameters of the prompt corrector (PC) sub-group (blue bars) of SMI patients compared to the non-prompt corrector (NPC) sub-group of SMI patients (yellow bars). The *p*_FS_, *p*_SS_, overall observed ingestion percent (OIP), excess dosing Poisson *λ*, von Mises mean, and von Mises concentration parameters are summarized as bar graphs in (**a–f**), respectively. The bars represent the mean value and the error bars are standard errors. The cumulative survival functions for the two groups’ observed ingestion duration (NPC: yellow line, PC: blue line) are compared in (**g**). The *p*-values for a–f are from a Mann-Whitney test and the *p*-value in g is from a log-rank test. For reference to text, Observed ingestion fraction = Observed ingestion percent/100

Figure 4 summarizes the mean digital medicine ingestion parameter profiles for the PC and NPC groups. The *p*_FS_ in the PC group was 1 by definition whereas the median *p*_FS_ in the NPC group was 0.5 (IQR = 0.296). The PC sub-group had higher *p*_SS_, overall OIP (median: 0.927 for PC vs. 0.700 for NPC), and von Mises concentration, as well as lower values of excess dosing event Poisson *λ* compared to the NPC group (for *p*-values see Fig. 4). The OIP, *p*_FS_, von Mises concentration and excess dosing event Poisson *λ* parameters are consistent with the PC group having a better overall digital medicine ingestion profile compared to the NPC group. The survival functions of the observed ingestion duration were similar between the two groups (Fig. 4g), suggesting that the PC group did not arise as a fragment of shorter durations on the system.

## Discussion

Digital medicine systems are providing near real-time access to objective medication ingestion information,^14^ enabling physicians and care teams to make more informed treatment decisions: Is medication being taken as prescribed? If there is a lack of clinical improvement, is medication adherence a factor? Objective digital medicine ingestion data will reduce the reliance on patient and caregiver reports that (psychiatric) care teams consider when addressing such treatment-related issues. The benefits of this data extend directly to the patient as well by eliminating subjectivity and perceptions of mistrust, both of which have previously demonstrated correlations with medication adherence in SMI.^18^

Digital medicine systems have begun to show benefit in clinical outcomes in patients with uncontrolled hypertension and type 2 diabetes.^10^ Improved patient engagement, effective monitoring, and the ability to make timely interventions were among the key drivers reported associated with the benefit derived from the digital medicine system. However, clinical efficacy is more challenging to measure in SMI where many of the available medications require weeks to months to elicit meaningful change, and there are no direct laboratory observations that can be associated with efficacy. In this context, objective data on dosing history may be the best direct measurement to inform clinical response, as it will enable distinction between patient- and drug-related treatement success factors on a more immediate timescale: Further, the ability to investigate variability in dose-timing, as well as patterns within periods of successful and unsuccessful dosing intervals will enable more personalized treatment recommendations.

In addition to leveraging digital medicine data within the context of a particular clinical visit or discussion with a patient, the scientific community must now also adopt new statistical frameworks—or re-evaluate and enhance existing ones—within the context of digital medicine to fully realize the benefits of this data, especially in the SMI patient population. With this in mind, we aimed to adapt and evaluate the performance of a Bayesian hybrid model to characterize digital medicine ingestion patterns in patients with SMI. Our results suggest that the hybrid Bayesian framework is a promising approach for characterizing medication ingestion behaviors obtained with a digital medicine system in SMI patients.

An observation of particular interest was the presence of a sub-group of Prompt Corrector (PC) patients who always successfully registered an ingestion following a failed ingestion event: This group comprised 22.1% of our sample. The presence of a subset with these desirable system characteristics was surprising. Part of our working hypothesis is that PC patients may be individuals who are well organized, follow a well-established routine for drug administration, and possibly other activities of daily living; however, we also found that more recently diagnosed patients were significantly more likely to be in the PC group, so there may also be a motivational component. It should be noted that within the current PC definition it is theoretically possible for a patient to take every alternate dose (50% expected ingestions observed) and still maintain the PC (*p*_FS_ = 1) criteria; however, systematically taking every alternate dose in such a fashion is likely a pre-planned and patterned choice that requires the patient to be highly conscientious about correcting missed doses and is not inconsistent with the notion of a prompt corrector. In long term dosing, beyond the currently explored 8-week digital medicine use-case, we acknowledge that the *p*_FS_ = 1 definition of prompt correctors may be too stringent; although as longer-term data sets become available, there may still be an appropriate *p*_FS_ threshold to classify PC patients with similar characteristics, and the ability to promptly correct missed ingestions.

These promising results in integrative modeling of ingestion patterns from a digital medicine system (DMS) represent a useful first step. However, additional high-resolution temporal data on other biometric markers such as actimetry and ECG that provide information on rest and activity patterns are also available with this DMS—these data streams could add additional contextual information for conditioning the priors in the Bayesian modeling framework. The framework is also versatile enough to accommodate distinctive sub-groups (such as the PC patients) by incorporating mixture models as priors in the Bayesian framework for the parameters. Further, the utility of contextual data outside the current digital medicine system should be evaluated, e.g., wearables and mobile passive sensing, potentially in conjunction with self-report ecological momentary assessments or brief clinical questionnaires.^19–21^

In conclusion, this work has demonstrated that a hybrid Bayesian modeling framework is capable of characterizing temporal patterns of successful and unsuccessful ingestion events from a digital medicine system in patients with serious mental illness. We have also identified immediate next steps and additional opportunities for research in the space. To our knowledge, this modeling framework is among the first to be applied to digitally acquired medication ingestion data—especially in the SMI population—and opens the door to new research possibilities in the area of medication adherence.

## Methods

### Digital medicine system (DMS)

The DMS consists of six primary components: (i) an ingestible event marker (IEM) embedded inside an active pharmaceutical; (ii) a patient-worn patch (on the torso); (iii) a mobile application; (iv) a secure cloud infrastructure for housing and making data available; (v) a care team portal that is accessible via a web browser; and (vi) a call-center to provide support to patients and their care teams. The patient-worn patch is designed for 7-days of wear and contains software to detect the IEM after ingestion, as well as a three-axis accelerometer, electrocardiogram and other sensors. All of the data that is collected from the patch is transmitted to the mobile application and then to the secure cloud infrastructure where it may be made available to appropriate members of the care team. Figure 1 provides a high-level overview of the DMS system.

This digital medicine system requires a patient-worn device to detect ingestions; however, we do not address the complexity of patch compliance in this work. We define a successful ingestion event as an observed ingestion, and an unsuccessful ingestion event as an unobserved ingestion. The “observed ingestion duration” is then defined as the elapsed duration between the first and last observed ingestion. As an analogue to overall adherence, we use the “observed ingestion percent” (OIP), defined as the fraction of ingestions observed within the observed ingestion duration.

### Clinical study descriptions

Both of these studies provided smartphones with the appropriate DMS software pre-loaded and required male and female patients to be on stable, once-daily, doses of oral aripiprazole: It was required that patients were deemed “capable” of using a DMS. During these studies patients received only the digital versions of their stable oral aripiprazole dose. Further, both studies received human subject approvals from the appropriate institutional review boards and subjects provided informed consent.

Study 1 was a multicenter, 8-week, open-label study with a primary objective of capturing the usability of the DMS by adult subjects with a diagnosis of schizophrenia with regard to their ability to independently (and successfully) replace their patch by the end of week 8 (NCT02219009). Patients were expected to perform five site visits following the screening period: baseline, and weeks 1, 2, 3, and 8.

Study 2 was a multicenter, 8-week, open-label, single-arm, exploratory trial with a primary objective of assessing the functionality of an integrated call center for the DMS by adult subjects with primary diagnoses of schizophrenia, major depressive disorder, or bipolar 1 disorder (NCT02722967). This study consisted of two phases: A 2-week prospective phase, and a 6-week observational phase. In order to progress to the 6-week observational phase, patients were required to have at least 50% patch data capture for the 7 days prior to the week 2 visit. Subjects who met this criterion were eligible to continue into the 6-week observational phase and would be expected to complete four total site visits (baseline and weeks 2, 4, and 8).

### Modeling digital medicine ingestion profiles

The hybrid Markov chain-von Mises model has been described in detail elsewhere.^12^ We recapitulate its key features here for completeness. The individual model consists of four inter-dependent components:

1. A two-state Markov chain was used to model the occurrence of unobserved ingestions (failures) and observed ingestions (successes).
2. The ingestion-timing deviations were modeled with a von Mises distribution.
3. Observed ingestion duration was modeled with a Weibull distribution.
4. The frequency of observed excess dosing events was modeled with a Poisson distribution.

#### Modeling the two-state Markov chain

The short-range dependence of ingestion observations was modeled using a two-state time-homogeneous Markov chain with transition matrix *A*:

$${\boldsymbol{A}} = \begin{array}{*{20}{c}} S \\ F \end{array}\left[ {\begin{array}{*{20}{c}} {p_{SS}} & {p_{SF}} \\ {p_{FS}} & {p_{FF}} \end{array}} \right] = \begin{array}{*{20}{c}} S \\ F \end{array}\left[ {\begin{array}{*{20}{c}} {p_{SS}} & {1 - p_{SS}} \\ {p_{FS}} & {1 - p_{FS}} \end{array}} \right]$$

The probability of a success (observed ingestion) following a success at the preceding dosing event was denoted by *p*_SS_, and the probability of a success following a failure (unobserved ingestion) at the preceding event was denoted by *p*_FS_.

Maximum-likelihood estimates^22^ of *p*_SS_ and *p*_FS_ were obtained from *N*_SS_, *N*_FS_, *N*_FF_, and *N*_SF_, the frequencies of success followed by success, failure followed by success, failure followed by failure and success followed by failure events, respectively, using:

$$\begin{array}{l}\hat p_{SS} = \frac{{N_{SS}}}{{N_{SS} + N_{SF}}}\\ \hat p_{FS} = \frac{{N_{FS}}}{{N_{FS} + N_{FF}}}\end{array}$$

The transition matrix *A*_*i*_ used for defining the Markov chain normalizes each row of *N*_*i*_ in subject *i*:

$${\boldsymbol{A}}_i = \begin{array}{*{20}{c}} S \\ F \end{array}\left[ {\begin{array}{*{20}{c}} {p_{SS}(i)} & {p_{SF}(i)} \\ {p_{FS}(i)} & {p_{FF}(i)} \end{array}} \right] = \begin{array}{*{20}{c}} S \\ F \end{array}\left[ {\begin{array}{*{20}{c}} {p_{SS}(i)} & {1 - p_{SS}(i)} \\ {p_{FS}(i)} & {1 - p_{FS}(i)} \end{array}} \right]$$

The parameterization of this model consisted of hyperbolic functions of two parameters, *s*(*i*) and *σ*(*i*), that underlie the helix-coil transition model in polymer physics,^15–17^ which has been previously explored as a viable model for adherence modeling.^13^

$${\boldsymbol{A}}_i = \left[ {\begin{array}{*{20}{c}} {\frac{{s\left( i \right)}}{{1 + s\left( i \right)}}} & {\frac{1}{{1 + s\left( i \right)}}} \\ {\frac{{\sigma (i)s\left( i \right)}}{{1 + \sigma (i)s\left( i \right)}}} & {\frac{1}{{1 + \sigma (i)s\left( i \right)}}} \end{array}} \right]$$

The priors for *s*(*i*) and *σ*(*i*) were:

$$\begin{array}{l}s\left( i \right) = e^{{\mathrm{z}}(i)}\\ z\left( i \right)\sim Normal(a_s,b_s)\\ \sigma (i)\sim Gamma(a_\sigma ,b_\sigma )\end{array}$$

Where z(*i*) is an intermediate dummy variable. *a*_*s*_ and *b*_*s*_ represent the mean and precision of the normal distribution, respectively, while *a*_*σ*_ and *b*_*σ*_ represent the shape and scale parameters of the Gamma distribution.

#### Modeling observed ingestion-timing deviations

The ingestion-timing deviation of the *i*^th^ ingestion, *δ*_*i*_, was defined as the difference between the actual ingestion time and the closest expected ingestion time. The probability density function (PDF) of the ingestion-timing deviations *δ* relative to *τ* (the dosing interval) after transformations to angular coordinates, *θ*, are assumed to be distributed according to the von Mises (VM) PDF function VM (*ψ*,*ω*):

$$\theta = 2\pi \frac{\delta }{\tau } \sim VM\left( {\psi ,\omega } \right)$$

The VM distribution describes angular random variables, and its PDF *p*(*θ*) at angular position *θ* radians is:

$$p\left( \theta \right) = \frac{{e^{\omega \ast cos\left( {\theta - {\mathrm{\psi }}} \right)}}}{{2\pi I_0\left( \omega \right)}}for\,\psi - \pi \le \theta \le \psi + \pi$$

The *ψ*(*i*) is the mean and *ω*(*i*) is a measure of how concentrated the ingestion-timing deviation angles are around the mean in subject *i*; *I*_*o*_ is the Bessel function of order zero.

The R circular statistics package^23^ was used to obtain maximum likelihood estimates for *ψ* and *ω* for each subject. The prior for the von Mises location parameter *ψ*(*i*) was a von Mises distribution. The prior for the von Mises concentration parameter *ω*(*i*) was assumed to follow a log-normal distribution.

$$\begin{array}{l}\psi (i)\sim vonMises(a_\psi ,b_\psi )\\ \omega (i)\sim LogNormal(a_\omega ,b_\omega )\end{array}$$

Where *a*_*ψ*_ and *b*_*ψ*_ are the same VM parameters as above, and the *a*_*ω*_ and *b*_*ω*_ represent the mean and precision of the log-normal prior, respectively.

### Modeling observed excess dosing events

In this analysis, an excess dosing event was defined as more than one ingestion observed in a given day for a given patient. The distribution of observed excess dosing events was modeled with a Poisson distribution. Maximum-likelihood estimation was used to calculate Poisson rate parameter *λ*(*i*) for subject *i*, whose prior in the population was assumed to follow an exponential distribution with rate parameter *a*_*λ*_:

$$\lambda (i)\sim Exponential(a_\lambda )$$

### Modeling observed ingestion duration

Observed ingestion duration was defined as the number of dosing intervals between the first observed ingestion and the last observed ingestion. For once-daily expected dosing, *N*_Total_[*i*], the total number of prescribed dosing events for the ingestion duration, would be numerically equivalent to the familiar time-on-treatment for subject *i*.

The total number of prescribed dosing events for the observed ingestion duration *N*_Total_[*i*] was modeled using a Weibull distribution with shape parameter *υ* and scale parameter *κ*:

$$N_{Total}[i]\sim Weibull\left( {\upsilon ,\kappa } \right)$$

The Weibull density, *Weibull*(*υ*,*κ*), with shape parameter *υ* and scale parameter *κ*, is defined with proportional hazards parameterization for a random variable *x* as:

$$Weibull\left( {\upsilon ,\kappa } \right) = x^{\upsilon - 1}e^{ - (x/\kappa )^\upsilon }$$

The Weibull parameters were estimated using the maximum-likelihood method in Mathematica (Wolfram Research, Champaign, IL). MCMC modeling was not used because *υ*, the Weibull shape parameter, shows poor mixing.^24^

### Markov chain Monte Carlo implementation

Mathematica (Wolfram Research, Champaign, IL) was used for estimating individual level parameters. Both SPSS (IBM, Armonk, NY) and the R statistics program were used for exploratory statistical analyses.^23,25^

Bayesian analysis was conducted using the well-established Markov Chain Monte Carlo (MCMC) method in the RJAGS software (Martyn Plummer). To allow the MCMC chains to converge we employed a conservative burn-in phase of 50,000 runs before evaluating any statistics. The MCMC algorithm was implemented for 3 chains each with 200,000 runs and thinning interval of 2.

The MCMC runs were analyzed with the CODA package.^26^ Add-on code for analyzing the von Mises distribution in RJAGS was developed by Colin Stoneking and Klaus Oberauer (Department of Cognitive Psychology, University of Zurich, Switzerland).

Convergence and mixing were assessed using multiple approaches. First, visual inspection of parameter trace plots was conducted for evidence of poor mixing. The Gelman-Rubin-Brooks plot, which shows the evolution of Gelman and Rubin’s shrink factor as the number of iterations increases,^27^ and the Gelman and Rubin multiple sequence convergence diagnostic were also examined.^27–29^ The Gelman-Rubin statistic was less than 1.05.

For visual predictive checks, the empirical density functions were computed from the histogram data. The density plots from RJAG simulations were compared to the empirical density functions. Histogram densities were visually overlaid on the estimated density function to obtain a visual reference.

The Bayesian analyses were conducted on a MacBook Pro laptop computer running the OS X Yosemite operating system version 10.10.3.

### Analyses of the prompt corrector sub-group

Following the identification of this sub-group of patients, we defined a patient to be a PC if they exhibited a *p*_FS_ = 1 and as a NPC otherwise. Mann-Whitney tests were used to compare model ingestion-related parameters of the PC group to those of the NPC group. The frequencies of PC patients in the schizophrenia, bipolar type 1, and major depressive disorder populations of SMI were compared using the *χ*^2^ test. The survival functions of the observed ingestion duration were compared using the Kaplan-Meier log-rank test. These analyses were conducted using IBM SPSS Statistics software (version 24, IBM Corp., Armonk, NY).

### Code Availability

Based on the proprietary nature of modifications made to the code to accommodate the digital medicine data, it may not be made available in all cases for a period of at least five years from publication. Requests for access to source code must be made on an individual basis to the corresponding author and would require evaluation on an individual basis. The authors made the appropriate materials available to the editorial staff during the review process for verification of results.

## Data Availability

Based on the proprietary nature of the data, it may not be made available for a period of at least 5 years from publication. Requests would require evaluation on an individual basis. The authors made the appropriate materials available to the editorial staff during the review process for verification of results.
